# Chemical Composition, Antioxidant and Cytoprotective Potentials of *Carica papaya* Leaf Extracts: A Comparison of Supercritical Fluid and Conventional Extraction Methods

**DOI:** 10.3390/molecules26051489

**Published:** 2021-03-09

**Authors:** Boon-Keat Khor, Nelson Jeng-Yeou Chear, Juzaili Azizi, Kooi-Yeong Khaw

**Affiliations:** 1 School of Pharmaceutical Sciences, Universiti Sains Malaysia, Minden 11800, Penang, Malaysia; khorboonkeat@hotmail.com; 2 Centre for Drug Research, Universiti Sains Malaysia, Minden 11800, Penang, Malaysia; jychear@gmail.com; 3 School of Pharmacy, Monash University Malaysia, Bandar Sunway 47500, Selangor Darul Ehsan, Malaysia

**Keywords:** *Carica papaya*, supercritical carbon dioxide, co-solvent, α-tocopherol, squalene, rutin, antioxidant activities, hydrogen peroxide, skin fibroblast

## Abstract

The leaves of *Carica papaya* (CP) are rich in natural antioxidants. *Carica papaya* has traditionally been used to treat various ailments, including skin diseases. This study aims to decipher the antioxidant effects and phytochemical content of different CP leaf extracts (CPEs) obtained using supercritical carbon dioxide (scCO_2_) and conventional extraction methods. The antioxidant activities of CPEs were evaluated by cell-free (1,1-diphenyl-2-picryl-hydrazyl (DPPH) and ferric-reduced antioxidative power (FRAP)) and cell-based (H_2_O_2_) assay. Both *C. papaya* leaf scCO_2_ extract with 5% ethanol (CPSCE) and *C. papaya* leaf scCO_2_ extract (CPSC) exhibited stronger DPPH radical scavenging activity than conventional extracts. In the FRAP assay, two hydrophilic extracts (*C. papaya* leaf ethanol extract (CPEE) and *C. papaya* freeze-dried leaf juice (CPFD)) showed relatively stronger reducing power compared to lipophilic extracts. Cell-based assays showed that CPFD significantly protected skin fibroblasts from H_2_O_2_-induced oxidative stress in both pre-and post-treatment. CPEE protected skin fibroblasts from oxidative stress in a dose-dependent manner while CPSCE significantly triggered the fibroblast recovery after treatment with H_2_O_2_. GC-MS analysis indicated that CPSCE had the highest α-tocopherol and squalene contents. By contrast, both CP hydrophilic extracts (CPEE and CPFD) had a higher total phenolic content (TPC) and rutin content than the lipophilic extracts. Overall, CPEs extracted using green and conventional extraction methods showed antioxidative potential in both cell-based and cell-free assays due to their lipophilic and hydrophilic antioxidants, respectively.

## 1. Introduction

The skin is the largest organ in the body and comprises the epidermis and dermis, which are formed by keratinocytes and fibroblasts [1]. The primary function of skin is to provide a protective barrier to prevent hazardous materials and pathogens from entering the body [2]. As the outermost layer of the body, the skin is directly exposed to environmental hazards and pollutants which are themselves oxidants or able to catalyze the production of reactive oxygen species (ROS) [3]. Reactive oxygen species (ROS) are a group of molecules which consist of free radicals (superoxide anion (O_2_^•−^), hydroxyl radical (OH^−^), and non-radical oxidants (hydrogen peroxide (H_2_O_2_)) [4]. Their sources can be varied and include environmental pollutants, UV radiation, and byproducts from cellular metabolism. ROS can be produced physiologically through the leaking of the electron from the mitochondria during oxidative respiration and as a byproduct of numerous enzymatic reactions such as those of NADPH oxidases [4]. At low levels, ROS act as a redox signaling messenger, regulating the physiological functions of the cells through activation of several antioxidant enzymes such as catalase, glutathione peroxidase, and superoxide dismutase [5]. However, imbalances in the production and removal of ROS cause oxidative stress, which is deleterious to the cells. ROS cause damage to the cell membrane through lipid peroxidation, mitochondrial dysfunction, and alterations in DNA and RNA structures, leading to apoptosis and cellular death [6]. Oxidative stress is a hallmark of many phenomena such as skin ageing, neurodegenerative diseases, and diabetic kidney [7,8,9]. Thus, maintaining the homeostasis between the oxidation and reduction of ROS is important in preventing diseases.

*Carica papaya* (CP) belongs to the family Caricaseae and is a perennial tree which is planted domestically for fruit production. Besides the fruit (which is used as a delicacy worldwide), the leaves have traditionally been used in treating parasitic worms, gastric digestion problems, fever, and burns, and for the relief of asthma [10,11,12]. Pharmacological studies have revealed that the leaves display antioxidant, anti-inflammatory, antiviral, antitumor, and antibacterial activities [13]. Our previous study showed that *C. papaya* leaf supercritical carbon dioxide (scCO_2_) extract (CPSC) and its constitutive phytosterols were cytotoxic towards squamous carcinoma cells (SCC25), a widely used model for skin cancer [14]. However, the effects of CPSC on non-cancerous skin cells are yet to be explored. In contrast, a study on human skin fibroblasts (HSF1184) revealed the wound-healing properties of CP leaf methanolic extract [15]. Besides the leaves, other parts of CP such as the seeds, latex and epicarp also exhibited wound-healing properties in in vitro and in vivo studies [16,17,18]. The exact mechanisms of CP’s wound healing potential are still obscure; however, diminished oxidative stress may play a role [19,20]. The leaves contain multiple bioactive components such as manghaslin, clitorin, rutin, nicotiflorin, papain, chymopapain, cystatin, α-tocopherol, ρ-coumaric acid, and caffeic acid that may contribute to the defense against oxidative stress [10,21,22]. 

In the pursuit of finding the best mixture of bioactive compounds through the extraction process, CP leaf extracts (CPEs) obtained from several extraction methods were suggested for testing in a targeted bioassay [14]. Conventional (maceration, soxhlet, juicing, and sonication) and non-conventional scCO_2_ green extraction methods have previously been employed to extract bioactive compounds from CP leaves [21,23,24,25]. The advantages of non-conventional scCO_2_ extraction include the need for little or no organic solvent, non-toxicity, and the resulting lack of solvent residues, making this extraction technique environmentally friendly [26]. However, scCO_2_ is only beneficial for the extraction of small non-polar molecules, while polar molecules remain unextracted. The incorporation of co-solvents such as ethanol or water during scCO_2_ extraction can resolve this problem and result in inclusion of polar molecules, thus enhancing extraction range of targeted bioactive compounds [26]. 

In this study, the phytochemical profiling of CPEs extracted by conventional and non-conventional green extraction was performed using GCMS and HPLC analysis. Following this, the antioxidant potential of CPEs was evaluated using cell-free and cell-based assays for the first time. Specifically, the cytoprotective effect of CPEs in non-cancerous Hs27 skin fibroblast cell lines was assessed under oxidative stress conditions.

## 2. Results

### 2.1. GC-MS Analysis of Lipophilic Constituents in CP Leaf Extracts 

**Figure 1:**
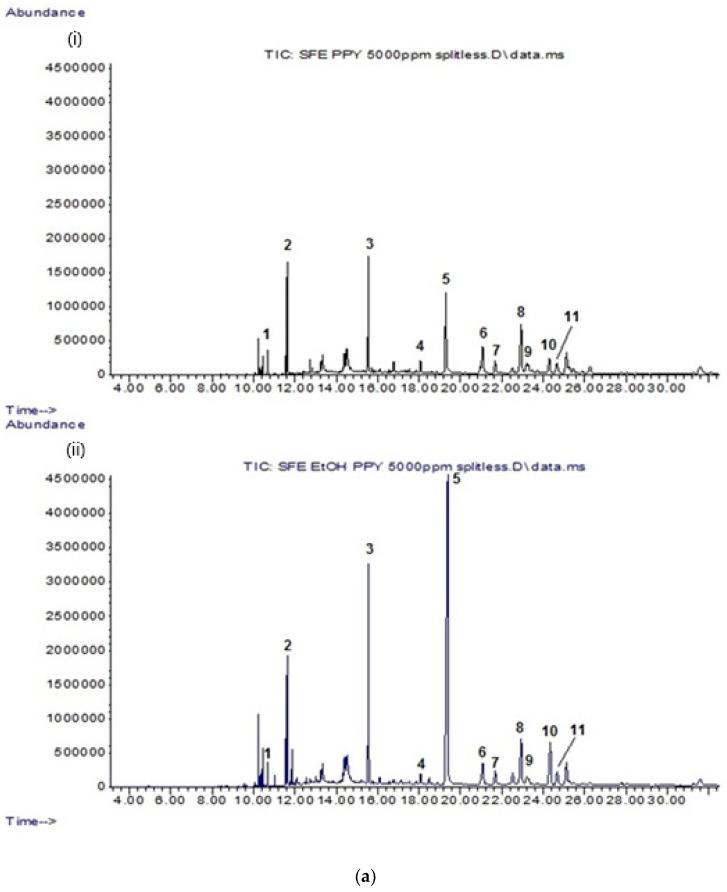

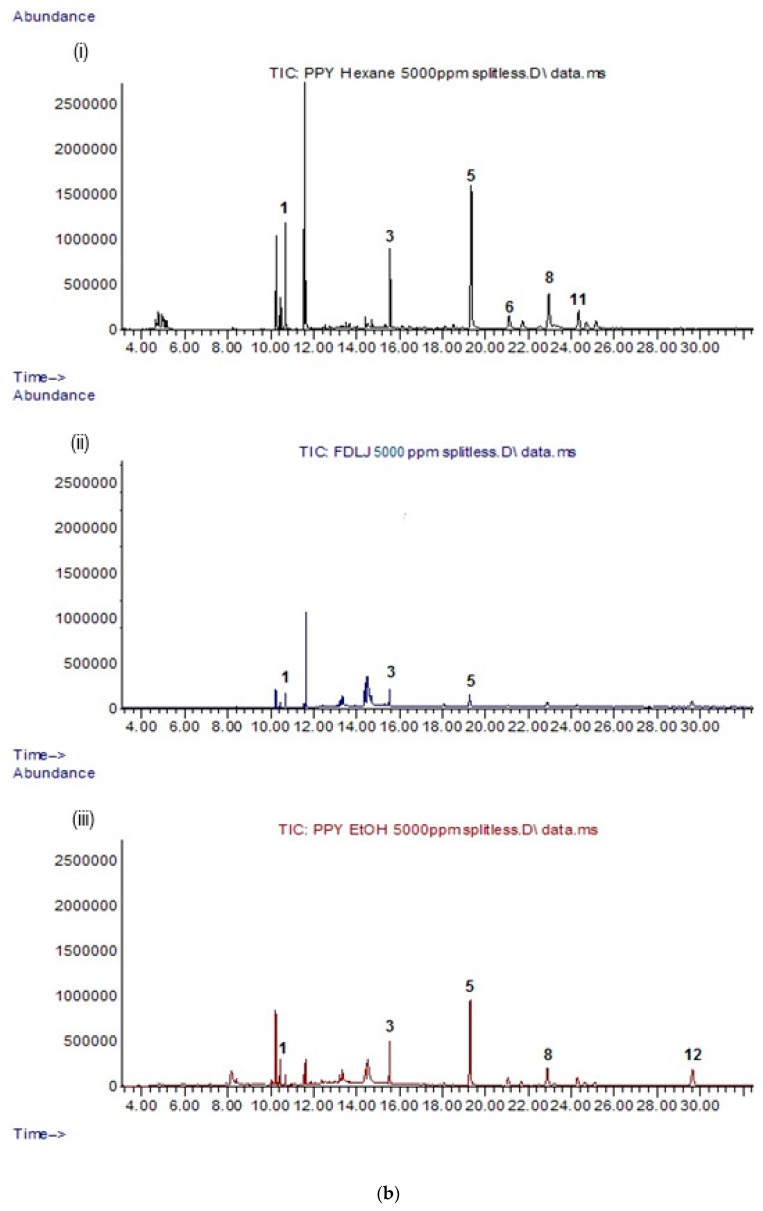
(**a**) Total ion chromatograms (TIC) of (i) CPSC extract; (ii) CPSCE extract at a fixed concentration of 5 mg/mL. The identified compounds (**1**–**11**) are listed in Table 1. (**b**) TIC chromatograms of conventional solvent extracts: (i) CPHE extract; (ii) CPFD extract; (iii) CPEE extract at a fixed concentration of 5 mg/mL. The identified compounds (**1**–**12**) are listed in Table 1. CPHE: *Carica papaya* leaf hexane extract; CPFD: *C. papaya* leaf juice freeze-dried extract; CPEE: *C. papaya* leaf ethanol extract; CPSC: *C. papaya* leaf scCO_2_ extract; CPSCE: *C. papaya* leaf scCO_2_ extract with 5% ethanol.

**Figure 2:**
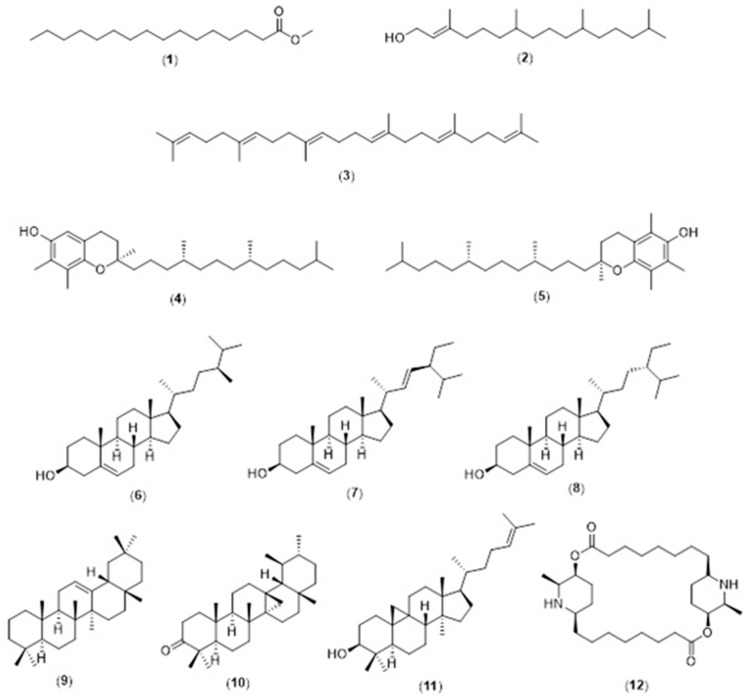
Chemical structures of identified lipophilic compounds (**1**–**12**) in *Carica papaya* (CP) extracts. The identified compounds are listed in Table 1.

All CP leaf extracts (5 mg/mL) resulting from both supercritical carbon dioxide and conventional extraction methods were evaluated for their lipophilic constituents using GC-MS. The details of the identified compounds are provided in Table 1. In general, CPSC and *C. papaya* leaf scCO_2_ extract with 5% ethanol (CPSCE) were found to contain a greater variety and number of lipophilic constituents as compared to the conventional leaf extracts (*C. papaya* leaf hexane extract (CPHE), *C. papaya* leaf ethanol extract (CPEE), and *C. papaya* leaf juice freeze-dried extract (CPFD)). CPSC and CPSCE were rich in lipophilic constituents such as essential fatty acids, phytosterols, and triterpenes (Figure 1a). By contrast, the conventional leaf extracts (CPHE, CPEE, and CPFD) contained relatively fewer constituents (Figure 1b). A total of 11 lipophilic constituents were identified in the CPSCE extract (Figure 1a). The identified major constituents were α-tocopherol (**5**), squalene (**3**), phytol (**2**), and a mixture of phytosterols such as campesterol (**6**), stigmasterol (**7**), and β-sitosterol (**8**), consistent with those reported in our previous study (Figure 2) [27]. A number of minor constituents such as hexadecanoic acid (**1**), γ-tocopherol (**4**), olean-12-ene (**9**), 13,17-cycloursan-3-one (**10**), and cycloartenol (**11**) were also detected in the CPSC extract. Interestingly, greater constituent concentration was observed in the CPSCE extract, which was extracted with supercritical fluid and a co-solvent (ethanol (5% *v*/*v*)). The extraction yields of the major constituents—(**3**) and (**5**)—in CPSCE were found to be 2- and 5-fold higher than those of the CPSC extract. Both CPSC and CPSCE extracts contained higher amounts and a greater variety of phytol (**2**), triterpenes (**9**, **10**), and phytosterols (**6**, **7**, **8**, **11**) compared to the conventional solvent extracts (CPHE, CPE, and CPFD), indicating the efficiency of supercritical fluids in extracting lipophilic constituents. In summary, the strength of extraction methods in extracting lipophilic constituents was as follows: CPSCE > CPSC > CPHE > CPEE > CPFD. One component that was found to be exclusive to CPEE extract was carpaine (**12**), a major papaya alkaloid [28].

### 2.2. HPLC Analysis of Hydrophilic Constituents in CP Leaf Extracts

**Figure 3:**
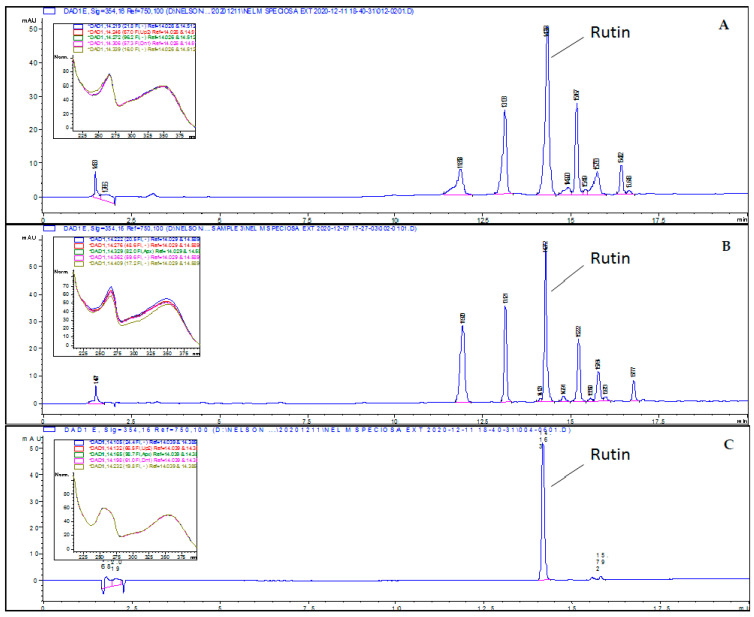
HPLC chromatogram of: (**A**) CPEE extract, (**B**) CPFD extract, and (**C**) the rutin standard at 14.2 min.

All the CPEs were subjected to HPLC-DAD analysis at a fixed concentration of 1 mg/mL following the programmed gradient method as described in Section 4.4. (b). Rutin, a major flavonoid found in CP leaves, was used as the chemical marker for quantification purposes [21,29]. However, only hydrophilic extracts (CPFD and CPEE) showed the separation (presence) of phenolic constituents in HPLC. The HPLC chromatograms for CPEE and CPFD extracts and rutin are given in Figure 3. The chromatographic peak of rutin in both extracts was identified by comparing its retention time, 14.27 min, with that of the rutin standard. The developed method was found to be selective due to its ability to separate rutin from other phytochemicals, and the targeted peak was free from interferences in the tested extract based on the UV spectrum (Figure 3). A linear calibration curve of rutin standard was established between 1.56 and 25.0 μg/mL, with a mean equation of:

(1) y = 39.626x + 17.179

and a correlation coefficient (R^2^) of 0.9996. CPEE extract was found to contain 22.07 ± 0.93 mg of rutin (per g of dry extract), which was approximately 3-fold higher than that of the CPFD extract (8.58 ± 0.12 mg per g of dry extract).

### 2.3. Antioxidant Activity of CP Leaf Extract

(a) 1,1-diphenyl-2-picryl-hydrazyl (DPPH) scavenging activity

To assess the free radical-scavenging ability of CP leaf extracts, DPPH assays were performed. Table 2 shows DPPH scavenging of CPEs. All CPEs demonstrated DPPH scavenging activity, with 50% inhibitory concentration (IC_50_) values ranging from 69.05 to 459.86 μg/mL. Among the CPEs, CPSCE had the most promising DPPH free radical scavenging activity, which was comparable with that of the standard compound butylated hydroxytoluene. On the contrary, CPHE had the lowest DPPH scavenging activity (6.6-fold lower than that of CPSCE).

(b) Total phenolic content (TPC)

**Figure 4:**
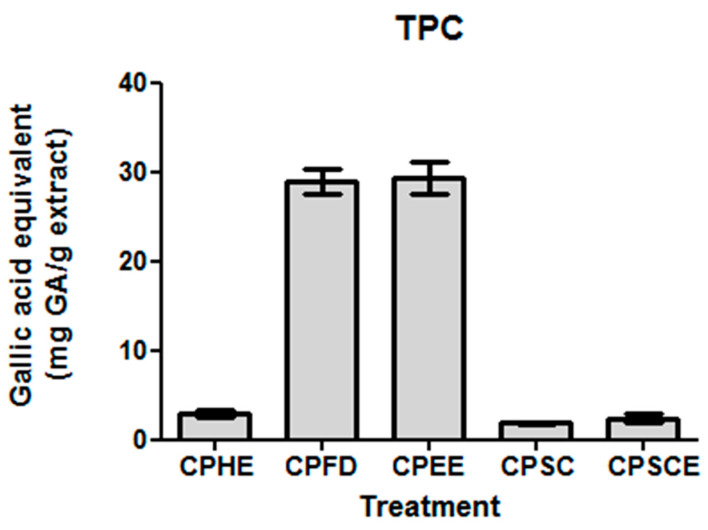
Total phenolic content of CPEs. The data are presented as the mean ± SEM of five independent experiments (*n* = 5).

The antioxidant properties for natural products are largely attributed to their total phenolic contents. Spectrophotometric assays were therefore employed to assess TPC levels. The TPC levels for CPEE and CPFD were 10 to 16-fold higher than those of CPHE, CPSE, and CPSC. CPEE (29.35 ± 1.72 mg gallic acid equivalents (GAE)/g) and CPFD (28.92 ± 1.43 mg GAE/g) showed the highest phenolic content, followed by CPHE (2.92 ± 0.41 mg GA/g), CPSCE (2.42 ± 0.46 mg GAE/g), and CPSC (1.85 ± 0.08 mg GAE/g). The results are presented in Figure 4.

(c) Ferric-reduced antioxidative power (FRAP)

**Figure 5:**
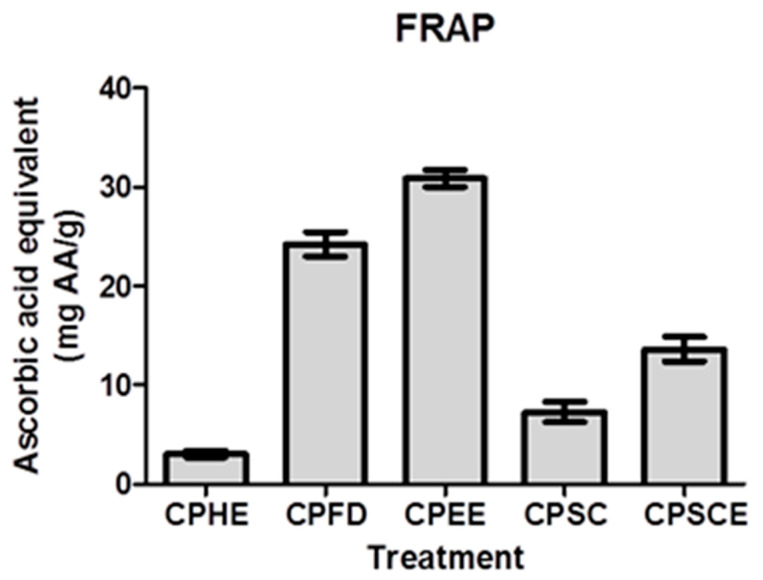
Ferric-reduced antioxidative power (FRAP) activity of CPEs. The data are presented as the mean ± SEM of five independent experiments (*n* = 5).

To further investigate the antioxidant potential of CPEs, FRAP assays were performed by which the assay was used to assess the reducing power of the samples (Figure 5). In this assay, CPEs with high reducing power reduced the Fe^3+^–2,4,6-tris(2-pyridyl)-s-triazine complex to a blue Fe^2+^–TPTZ complex. CPEE exhibited the highest FRAP reducing power at 30.88 ± 0.87 mg ascorbic acid equivalents (AAE)/g, followed by CPFD (24.24 ± 1.21 mg AAE/g), CPSCE (13.63 ± 1.24 mg AAE/g), CPSC (7.29 ± 1.03 mg AAE/g), and CPHE (3.04 ± 0.33 mg AAE/g).

### 2.4. Cytotoxicity of CP Leaf Extracts to Hs27 Human Skin Fibroblasts

**Figure 6:**
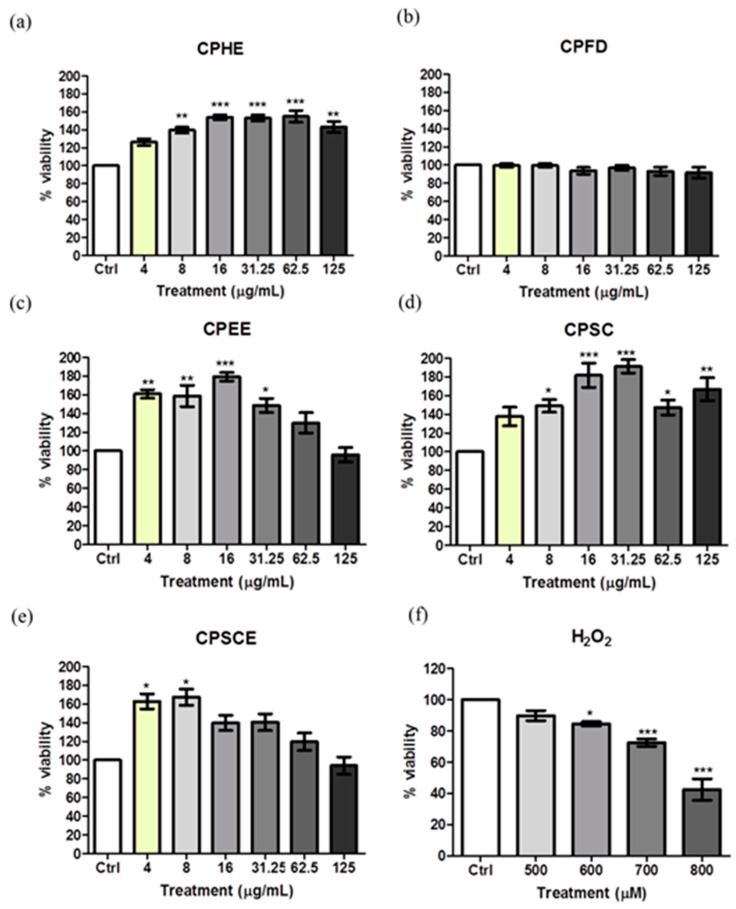
The viability of Hs27 fibroblast cells after exposure to different concentrations of extracts and H_2_O_2_. (**a**) CPHE, (**b**) CPFD, (**c**) CPEE, (**d**) CPSC, (**e**) CPSCE, and (**f**) H_2_O_2_. The data are presented as the mean ± SEM (*n* = 6). The significant difference between the groups was calculated using one-way ANOVA with Tukey’s post-hoc test. * represents *p* < 0.05, ** represents *p* < 0.01, and *** represents *p* < 0.001 as compared to the control.

Hs27 human skin fibroblasts were employed to assess the effect of CPEs on cell viability. As shown in Figure 6, none of the CPEs (4 to 125 μg/mL) exhibited significant toxicity to skin fibroblasts after the 48-h incubation period. All CPEs triggered fibroblast proliferation except for CPFD. CPHE, CPSC, CPSCE, and CPEE showed a proliferation-enhancing effect between 4 to 125 μg/mL. This indicates that the safety and therapeutic range of *C. papaya* leaf extracts was below 125 μg/mL. Thus, treatment doses between 25 to 100 μg/mL were selected for the pre- and post-treatment assays. Cells treated with H_2_O_2_ showed a significant dose-dependent reduction in viability. Cells treated with 700–800 μM of H_2_O_2_ resulted in a fibroblast killing effect of between 30% and 60%. Based on calculations, the IC_50_ value of H_2_O_2_ was approximately 750 µM. Thus, in the following assay, 750 μM H_2_O_2_ was used to induce 50% cell toxicity in the pre- and post-treatment assays.

### 2.5. Protective Effect of CP Leaf Extracts towards H_2_O_2_-Induced Oxidative Damage

**Figure 7:**
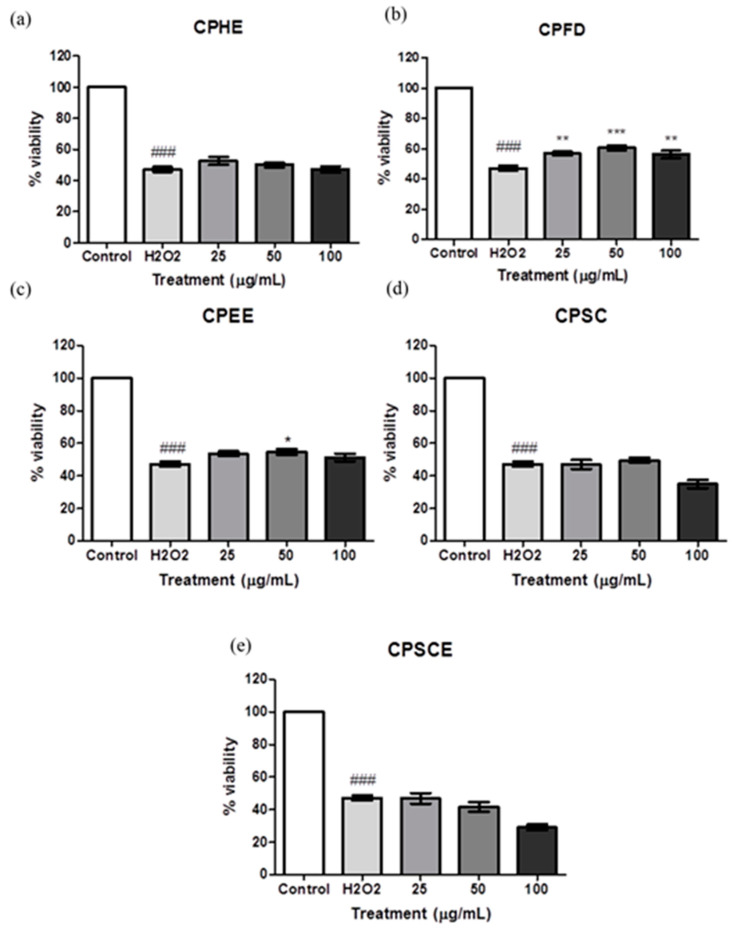
The viability of Hs27 fibroblast cells pre-treated with (**a**) CPHE, (**b**) CPFD, (**c**) CPEE, (**d**) CPSC, and (**e**) CPSCE after exposure to H_2_O_2_. The data are presented as means ± SEM (*n* = 6). The significant difference between the groups was calculated using one-way ANOVA with Tukey´s post-hoc test. * represents *p* < 0.05, ** represents *p* < 0.01 and *** represents *p* < 0.001 compared to H_2_O_2_; ### represents *p* < 0.001 compared to the control.

The antioxidant effect of the CPEs in the cell model was evaluated using the 3-(4,5-dimethylthiazol-2-yl)-2,5-diphenyl tetrazolium bromide (MTT) assay. Figure 7 shows Hs27 cell viabilities when exposed to CPEs. The CPEs were pre-treated with CPEs for 24 h, followed by another 24-h period of exposure of H_2_O_2_. Incubation of H_2_O_2_ in fibroblast cells reduced the cell viability in a dose-dependent manner. After exposure of 750 μM H_2_O_2_, cell viability was reduced to 50% as compared to untreated cells (100% viability). Meanwhile, pre-treatment with CPFD extract (ranging from 25 to 100 μg/mL) protected cells from oxidative damage significantly. Cells pre-treated with CPEE at 50 μg/mL showed statistical significance with regard to the proliferation of Hs27 cells. CPHE, CPSC, and CPSCE did not have promising effects on H_2_O_2_ induced oxidative damage. 

**Figure 8:**
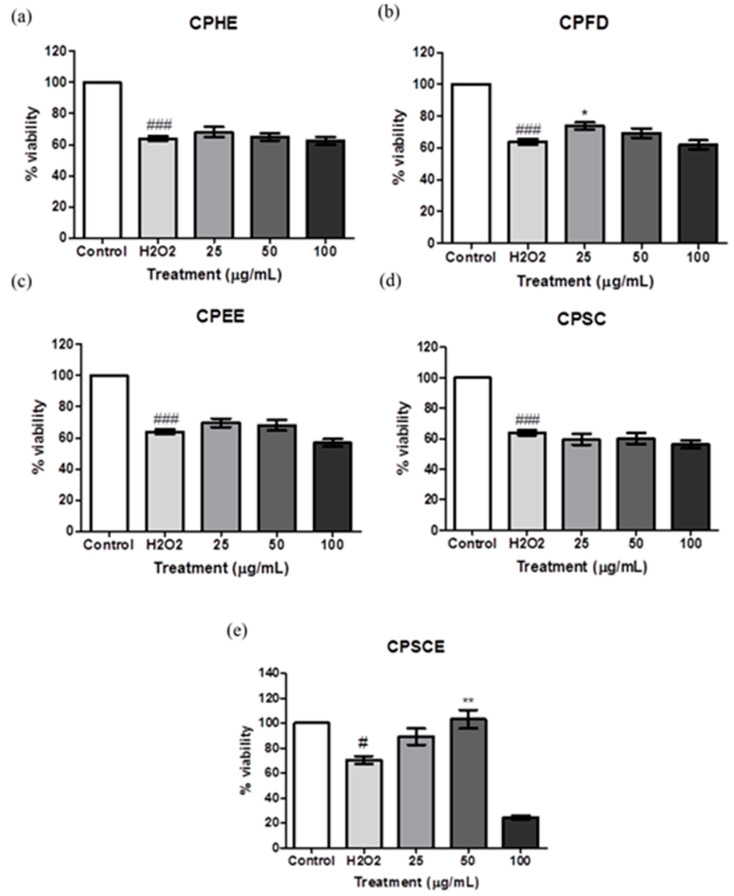
The viability of Hs27 fibroblast cells post-treated with (**a**) CPHE, (**b**) CPFD, (**c**) CPEE, (**d**) CPSC and (**e**) CPSCE after being exposed to H_2_O_2_. The data are presented as means ± SEM (*n* = 6). The significant difference between the groups was calculated using one-way ANOVA with Tukey´s post-hoc test. * represents *p* < 0.05, and ** represents *p* < 0.01 compared to H_2_O_2_; # represents *p* < 0.05, and ### represents *p* < 0.001 compared to the control.

The ability of CPEs with regard to cell recovery from oxidative damage by H_2_O_2_ on skin fibroblasts was evaluated by the MTT assays. CPEs (25 to 100 ug/mL) were introduced after the exposure to 750 μM H_2_O_2_ for 24 h. Figure 8 shows Hs27 cell viabilities when exposed to CPEs. CPFD and CPSCE at concentrations of 25 μg/mL and 50 μg/mL showed statistical significance in the proliferation of Hs27 cells when exposed to H_2_O_2_-induced oxidative stress. Approximately 10% of cells proliferated after treatment with 25 to 100 μg/mL of CPFD. Interestingly, up to 30% of Hs27 cells proliferated after treatment with 50 μg/mL of CPSCE. 

## 3. Discussion

The potential use of CP leaves in therapeutic skin care has been of great interest due to its abundance of natural antioxidants such as vitamin E, phenolics, flavonoids, etc. Previous reports revealed that CP leaf extract improved wound healing in in vivo models and exhibited a protective effect in the skin against UV radiation [30,31]. However, the phytochemicals which are responsible for the skin protection have not been well-identified. In this study, green and conventional extraction methods were employed to extract the bioactive compounds and evaluate their potential antioxidant and skin-protective effects. 

Our results showed that scCO_2_ extraction of CP leaves with 5% of ethanol as a co-solvent increased lipophilic bioactive compounds, as revealed by GCMS analysis. The addition of a small amount of ethanol or methanol as co-solvent is encouraged to lower the activation energy and enhance the transportation of metabolites to the fluid, thus increasing the extraction yield [26]. Consistent with this theory, the α-tocopherol and squalene contents extracted by scCO_2_ + 5% ethanol (CPSCE) were 5- and 2- fold higher than scCO_2_ (CPSC) alone and extracts extracted by conventional extraction methods. In addition, the extraction yield of CPSCE extracted by scCO_2_ + co-solvent was 150% higher than that of CPSC extracted by scCO_2_ alone. Interestingly, the scCO_2_ extraction yield of papaya leaves was 1.5–3 fold lower than those found in previous study, despite the use of an additional co-solvent [27]. It might be reasoned that in different geographical locations the soil pH promotes the biosynthesis of bioactive compounds. Next, all CPEs were evaluated for their antioxidant and cytoprotective potentials in vitro. 

The potential of an extract in attenuating oxidative stress is based on two main criteria: (1) the scavenging ability and (2) the possibility of activation of oxidative genes. In this study, the antioxidant capability of CPEs was evaluated by DPPH, FRAP, and TPC. CPSCE had the highest free radical scavenging activity as compared with CPSC, CPHE, and CPFD. This might be attributed to the presence of the α-tocopherol as a major component in the CPSCE. α-tocopherol is a well-known antioxidant exhibiting excellent scavenging activity and cytoprotection in various in vitro and in vivo oxidative stress models [32,33,34]. Besides, the presence of phytosterols in the lipophilic extracts might enhance the total radical scavenging activity through the formation of an allylic free radical. This allylic radical will then isomerize the existing radicals to other relatively stable free radicals [35]. Interestingly, CPSCE also significantly improved (~30%) the recovery of Hs27 cells after treatment with hydrogen peroxide. This can be attributed to the presence of active secondary metabolites including squalene and α-tocopherol in CPSCE for cell regeneration [33]. In addition, topical application of α-tocopherol and squalene have been reported to improve wound healing and skin regeneration [36,37,38].

On the other hand, phenolic-rich extracts (CPFD and CPEE) exhibited stronger ferric-reducing activity, with moderate protective effects against H_2_O_2_-induced oxidative stress in CPE-pretreated skin fibroblasts. The antioxidant capacity of phenolic compounds is mainly due to their redox properties. These properties allow them to act as excellent reducing agents, hydrogen donors, singlet oxygen quenchers, or metal chelators. Hypothetically, the structure of phenolic compounds, which consists of hydroxyl groups in ring B and the presence of carbon 2 and carbon 3 double bonds connected with the carbon 3 hydroxyl group and carbon 4 carbonyl group are important in both reducing power and radical scavenging effect. These essential structures are found in bioactive components such as rutin and phenolic acids in hydrophilic extracts (CPFD and CPEE) [39,40,41,42]. Through HPLC analysis, rutin (a flavonol glycoside) was found to be one of the major flavonoids in CPFD and CPEE, in accordance with the literature [20,43]. Rutin is well known for its wound-healing and antioxidant properties, which may partly explain the reason why CPFD and CPEE enhanced fibroblast proliferation after post-treatment with H_2_O_2_ [44,45]. Rutin has been proven to enhance the production and accumulation of extracellular matrices during the fibroblast healing process [44]. However, the possible skin-protective effects of other unidentified constituents remain unknown and inconclusive. Besides, plant primary metabolites such as polysaccharides and peptides might also contribute to the total antioxidant activities of CPFD and CPEE in skin fibroblast protection (pretreatment). In addition, a previous study showed that polysaccharides from *Alfafa* and *Tremella fuciformis* improved survival and reduced oxidative stress in skin fibroblast cells [46,47]. 

## 4. Materials and Methods

### 4.1. Chemical and Reagent

Rutin hydrate (HPLC grade; purity >94%), and 3-(4,5-dimethylthiazol-2-yl)-2,5-diphenyltetrazolium bromide (MTT) were purchased from Sigma-Aldrich (St. Louis, Missouri, United States). Formic acid (98–100%), acetonitrile (HPLC grade), methanol (HPLC grade), hexane (analytical (AR) grade), and ethanol (AR grade) were purchased from Merck (Darmstadt, Germany). High-glucose Dulbecco’s Modified Eagle’s Medium (DMEM), trypsin, penicillin/streptomycin, and fetal bovine serum (FBS) were obtained from Invitrogen (Life Technologies, Mulgrave, VIC, Australia). The human skin cell line (Hs27 ATCC CRL-1634) was purchased from the American Type Culture Collection (Manassas, VA, USA).

### 4.2. Plant Materials

In total, 10 kg of fresh mature *C. papaya* leaves were collected from a papaya tree growing in a local fruit farm at Muar, Johor, Malaysia. The collected leaves were washed with tap water to remove contaminants. The leaf material was then separated into two portions, where (a) air-dried leaves were used for the extraction with hexane (CPHE), ethanol (CPEE), and supercritical fluids (CPSC and CPSCE), and (b) fresh leaves were used for leaf juice (CPFD) extraction. The air-dried leaf material was kept in an air-tight container and stored at −20 °C prior to extraction procedures.

### 4.3. Extraction Methods

(a) Supercritical fluid extraction

The scCO_2_ extraction of air-dried CPL was performed using Supercritical Fluid Preparative scale CO2 extraction equipment (Taiwan Supercritical Technology), and extraction parameters in accordance with a previously optimized method [27]. The scCO_2_ extraction was operated with the following parameters: pressure 250 bar, temperature 35 °C, extraction time: 3 h, and with or without 5% ethanol as co-solvent. All extracts were kept in a freezer (−20 °C) until further experimentation. The extraction yields for CPSC and CPSCE were 1.12 and 1.54%, respectively.

(b) Maceration

In total, 2 kg of air-dried CPL were extracted with hexane and ethanol, respectively, according to the procedures described previously [14]. All extracts were kept in a −20 °C freezer until further evaluation. The extraction yields of hexane and ethanol extracts were approximately 5.2 and 15.6%, respectively.

(c) Leaf juice extraction

In total, 2 kg of fresh CPL were blended with a juice extractor (Panasonic, Kobe, Japan). The leaf juice was filtered and then freeze-dried to produce the lyophilized leaf juice extract (CPFD). The CPFD was kept at −20 °C until further experimentation. The overall extraction yield was about 14%. 

### 4.4. Chemical Analysis

(a) GC-MS analysis

Lipophilic constituents of *C. papaya* extracts were determined by a hyphenated Agilent 6890N Network GC system coupled to an Agilent 5973i mass selective detector (Agilent Technologies, Germany) as described previously by Chear et al. (2016) [48]. An aliquot of 10 mg/mL extract (in methanol) was separated on a HP-5MS column (30 m × 0.25 mm, 0.25-µm film thickness; Agilent Technologies, Waldbronn, Germany) with helium gas flowing at 1.2 mL/min. The injection volume was 1 µL with a splitless mode. The initial column temperature was set at 70 °C for 2 min, and then slowly increased to 280 °C at a constant rate of 20 °C/min. The column temperature was maintained at the final temperature of 280 °C for another 20 min. The total run time was 32.5 min. The injector, detector, and interface temperatures were set at 250 °C, 280 °C, and 300 °C, respectively. Mass acquisition was performed in the range of 40–550 *m*/*z* using electron impact ionization at 70 eV. The detected compounds were identified by performing spectral database matching against the National Institute of Standards and Technology database (NIST 02; Gaithersburg, MD, USA). The identity of the detected compound was determined by comparing the mass of their molecular ions, base ions, and fragment ions, as well as their peak intensities with those reference standards in the database. The detected compounds with >90% spectral matching quality were considered acceptable.

(b) HPLC profiling of the polar extracts

A simple and selective high-performance liquid chromatography (HPLC) method was developed and validated for the analysis of CPL extracts. The HPLC analysis was performed on an Agilent 1200 series HPLC system coupled to a photodiode array detector (Agilent, Santa Clara, CA, USA). Briefly, a stock solution of CPL sample was prepared at 500 µg/mL in a mixture of methanol and water (80:20 *v*/*v*) and centrifuged to remove the undissolved particles. The chromatographic separation was achieved on an Eclipse C18 reversed phase column (4.6 mm × 150 mm, 3.5 µm) (Agilent Technologies, Santa Clara, CA, USA) at an adjusted temperature of 30 °C. The employed mobile phase was a mixture of 0.1% formic acid in water (pH 2.65) (A) and acetonitrile (B) running at a gradient method with a flow rate of 1 mL/min. The detailed gradient method is provided in Table 3. Twenty-five microliters of CPL extract were injected to the system, and the total run time was 30 min. The detection and quantification of a major flavonoid marker (rutin) were achieved using an Agilent photodiode array detector at λ_max_ of 354 nm. Identification of rutin was done by comparing the HPLC retention time and UV spectrum of the analyte with that of reference standard. ChemStation LC3D software (Rev. B.03.01 317) was used for the data analysis. A 1000 μg/mL stock solution of rutin standard was prepared in methanol and then diluted into a series of 5 working standard solutions of 1.56, 3.125, 6.25, 12.5, and 25 μg/mL. Calibration curve was constructed by plotting the peak area against its corresponding concentration. Each individual standard/extract at a fixed concentration was consecutively injected three times.

### 4.5. Antioxidant Assay

(a) DPPH scavenging activity

The DPPH radical scavenging activity of the CPEs was evaluated using prior references [49,50]. The 1,1-diphenyl-2-picryl-hydrazyl (DPPH) radical scavenging capacity assay was determined by the decolourisation of the DPPH solution. Ten microliters of each sample with final concentrations in the range of 25 to 1000 ug/mL and 25 to 200 ug/mL for the standard were added to 96-well microtiter plate, followed by the addition of DPPH solution. Upon reaction in the dark for 30 min, the optical density of the solution was evaluated at 517 nm. The assay was done in triplicate.

(b) Total phenolic content

The total phenolic content (TPC) was performed using the Folin-–Ciocalteu method [51]. A total of 20 μL of 5 mg/mL CPEs in 25% (*v*/*v*) DMSO were mixed with 100 μL of 1:4 diluted Folin–Ciocalteu reagent and shaken for 1 min in a flat-bottom 96-well microplate. The mixture was left for 2 min followed by the addition of 75 μL of sodium carbonate solution (100 mg/mL) and the mixture was shaken at medium-continuous speed for 1 min. After 2 h at room temperature, the absorbance was measured at λ = 750 nm. The absorbance of the same reaction with water instead of the extract was subtracted from the absorbance of the reaction with CPE. Gallic acid (0.05–0.5 mg/mL) in 25% (*v*/*v*) DMSO were used as standards for calibration. The TPC was calculated as gallic acid equivalents (GAE) in mg per g of CPE (mg GAE/g).

(c) Ferric-reducing antioxidant power (FRAP)

The ferric reducing antioxidant power (FRAP) of CPEs were quantified using the method proposed previously [52]. An aliquot of 280 µL of the freshly prepared FRAP reagent and 20 µL of 1 mg/mL CPE in 5% DMSO were added to each well, and after 30 min reaction the absorbance was read at λ = 593 nm. The FRAP reagent was prepared fresh by mixing sodium acetate buffer (300 mM, pH 3.6), a solution of TPTZ (10 mM) in 40 mM HCl, and 20 mM FeCl_3_.6H_2_O using the proportion 10:1:1 (*v*/*v*/*v*). An analytical curve with different concentrations of ascorbic acid (0.01–0.1 mg/mL) was plotted to quantify the ferric reducing antioxidant power of the selected extracts. The results were expressed in mg ascorbic acid equivalents (AAE) per g of CPE (mg AAE/g).

### 4.6. Cell Culture and Maintenance

Hs27 skin fibroblasts were cultured in Dulbecco’s Modified Eagle’s Medium high glucose (ATCC) supplemented with 10% fetal bovine serum and 1% penicillin-streptomycin at 37 °C incubator supplied with 5% CO_2_. 

### 4.7. Cytotoxicity of C. papaya Extracts in Hs27 Human Skin Fibroblasts

MTT assay was used to evaluate the competency of CPEs. In brief, 5000/100 µL of Hs27 cells were seeded into a 96-well microtiter plate and incubated for 24 h. After that, the medium of each well was removed and replaced with CPEs prepared in various concentrations (4, 8, 16, 31.25, 62.5, and 125 µg/mL). The treated cells were incubated for 48 h at 37 °C in an incubator with 5% CO_2_. All CPEs were prepared in DMEM medium with reduced serum (1% FBS). For the growth control group, Hs27 cells were maintained in DMEM medium with 1% FBS. After 48 h, 10 μL MTT solution was added into each well. After 4 h of incubation, the medium in each well was removed and added with 100 μL DMSO to access cell viability. The optical density (OD) of each well was evaluated at 570 nm adjacent to the reference wavelength of 650 nm. Cell viability was calculated using the formula below:

(2) $$Percentage\;viable\;cell(\%)=\frac{Absorbance(Treated)-Absorbance(Blank)}{Absorbance(Untreated)-Absorbance(Blank)}\times 100\%$$

### 4.8. Protective Effect of C. papaya Leaf Extracts Against H_2_O_2_ Induced Toxicity

The protective studies of CPEs towards H_2_O_2_-induced oxidative stress were carried out as reported previously [53]. Hs27 human skin fibroblasts were seeded in 7.5 × 10^3^ cells per well in a 96-well plate and allowed to grow until a monolayer formed. To determine the protective effects of extracts, cells were either pre-treated or post-treated with CPEs. For the pre-treatment assay, cells were initially treated with different doses of extracts (25, 50, and 100 μg/mL) for 24 h, and then discharged and replaced with medium containing H_2_O_2_ (750 μM, IC50 of H_2_O_2_) for another 24 h.

For post-treatment assay, cells were treated with H_2_O_2_ (750 μM) for 24 h to induce oxidative damage. After that, the medium containing H_2_O_2_ was removed, and fresh medium containing different doses of extracts (25, 50, and 100 μg/mL) was added into the wells for another 24 h. The viability of the cells was determined by MTT assay. MTT was added to each well and subsequently incubated for 3 h. Upon incubation, the medium was removed and DMSO was added to dissolve the formed tetrazolium salt. The absorbance was measured at 490 nm by a Multiskan Go UV microplate reader. Cell viability was calculated as previously mentioned. 

## 5. Conclusions

Overall, scCO2 with the addition of co-solvent was shown to improve the α-tocopherol and squalene contents and total extraction yield. In addition, CPSCE showed the most potent free radical-scavenging activity in DPPH assay, while also protecting Hs27 cells against H_2_O_2_-induced cytotoxicity in the post-treatment assay. These activities are mainly attributed to its high α-tocopherol content. On the other hand, CPEE and CPFD exhibited significant reducing power in FRAP assay, and CPFD moderately protected Hs27 cells in pre- and post-treatment assays due to its high phenolic content (particularly rutin). However, future studies to investigate the wound-healing effect of single compounds from these extracts are warranted for the better understanding of the underlying skin-protective mechanisms. 

## Figures and Tables

**Table 1:** Relative amounts of lipophilic constituents in CP extracts.

Peak Label	Identified Compound	Retention Time (min)	* Corresponding % Maximum Based on Peak Area				
			CPHE	CPFD	CPEE	CPSC	CPSCE
1	Hexadecanoic acid	10.68	100	12.97	11.52	39.60	33.97
2	Phytol	11.61	ND	ND	ND	78.49	100
3	Squalene	15.55	27.15	4.88	13.72	54.19	100
4	γ-tocopherol	18.07	ND	ND	ND	100	97.76
5	α-tocopherol	19.29	26.42	2.11	14.61	19.43	100
6	Campesterol	21.07	37.56	ND	ND	100	78.89
7	Stigmasterol	21.67	ND	ND	ND	85.10	100
8	β-sitosterol	22.93	54.39	ND	26.33	100	93.53
9	Olean-12-ene	23.19	ND	ND	ND	100	ND
10	13,17-cycloursan-3-one	24.30	ND	ND	ND	33.89	100
11	Cycloartenol	24.66	86.35	ND	ND	78.97	100
12	Carpaine	29.65	ND	ND	100	ND	ND

* Percentage of peak area relative to the largest peak for each compound, which is set at 100%. ND = not detected.

**Table 2:** 50% inhibitory concentration (IC_50_) of DPPH scavenging activity of CPE.

CP Leaf Extract	IC_50_ (μg/mL)
CPHE	459.86 ± 11.91
CPFD	398.03 ± 31.84
CPEE	151.36 ± 4.38
CPSC	92.32 ± 3.58
CPSCE	69.05 ± 11.47
Butylated hydroxytoluene (standard)	89.1 ± 0.90

**Table 3:** Mobile phase gradient program.

Time	% A (0.1% Formic Acid)	% B (Acetonitrile)
5.0	90	10
20.0	70	30
22.0	5	95
25.0	5	95
26.0	90	10
30	90	10

## Data Availability

Not applicable.

## Sample Availability

Samples of the compounds are available from the authors.

## Abbreviations

*Carica papaya* leaf extracts (CPEs); *Carica papaya* leaf hexane extract (CPHE); *Carica papaya* leaf juice freeze-dried extract (CPFD); *Carica papaya* leaf ethanol extract (CPEE); *Carica papaya* leaf scCO_2_ extract (CPSC); *Carica papaya* leaf scCO_2_ extract with 5% ethanol (CPSCE).
